# Methods to promote seed germination in the lacquer tree, *Toxicodendron vernicifluum* (Stokes) F.A. Barkley

**DOI:** 10.1371/journal.pone.0272665

**Published:** 2022-08-11

**Authors:** Keiichirou Nemoto, Aiko Watanabe, Chiharu Yoshida, Masahiro Nishihara

**Affiliations:** Iwate Biotechnology Research Center, Kitakami, Iwate, Japan; University of Tsukuba, JAPAN

## Abstract

The lacquer tree, *Toxicodendron vernicifluum*, is a common industrial crop in East Asia. However, *T*. *vernicifluum* seeds are extremely difficult to germinate, which poses a major obstacle to establishing seedlings for sap production. In this study, we examined the germination properties of *T*. *vernicifluum* seeds in order to establish an inexpensive and effective method to promote seed germination. The seeds are covered with a hard endocarp, which we degrade using conventional sulfuric acid-based methods. Although sulfuric acid was effective in promoting seed germination, the germination rate was less than 5%. In addition to treatment with sulfuric acid, co-treatment with cold temperatures or the phytohormone gibberellic acid increased the germination rate to 22–35%. Seed viability analysis combined with specific gravity-based seed selection revealed that more than half of the seeds housed embryos that were incapable of germination. In additions, specific gravity-based seed selection aided in the selection of seeds capable of germination and improved the germination rate to approximately 47%. Taken together, our results suggest that the low germination rate of *T*. *vernicifluum* seeds is due to deep seed dormancy—which is controlled by physical and physiological mechanisms—and low embryo viability. To improve the germination rate of *T*. *vernicifluum* seeds, we propose an effective method whereby seeds with good germination capacity are selected based on specific gravity, following which their physiological dormancy is inactivated through cold pretreatment.

## Introduction

*Toxicodendron vernicifluum* (Stokes) F.A. Barkley, commonly known as the lacquer tree, is a deciduous tree that grows naturally in China and the Indian subcontinent [1]. In China, Korea, and Japan, the sap and fruits of *T*. *vernicifluum* have long been used in the production of candles, cosmetics, traditional herbal medicines, and highly durable coatings [2, 3]. In Japan, the sap of *T*. *vernicifluum* is known as “urushi”. Urushi has been used as a coating and adhesive substance for approximately 9,000 years [4], and its use is associated with a unique culture with historic and artistic value. In 2020, traditional techniques and knowledge of wooden construction involving the use of urushi were added to the United Nations Educational, Scientific and Cultural Organization’s representative list of Intangible Cultural Heritage of Humanity (https://ich.unesco.org/). The Agency for Cultural Affairs recommends the use of domestic urushi for the restoration of culturally important objects. Although the yearly requirement for urushi is estimated to be 2.2 tons or more, its annual production volume is only approximately 1.8 tons [5]. Therefore, the production cost of urushi has risen in recent years, exceeding 50,000 Japanese yen per kilogram [5]. Since urushi is also used in traditional handicrafts, there is rising concern that the soaring prices of urushi will affect the production and consumption of these crafts.

The traditional Japanese method of sap collection involves collecting sap until the tree dies, after which the tree is cut down. However, *T*. *vernicifluum* seedlings have to be grown for more than 10 years before the sap can be collected. Therefore, to increase the production of urushi, it is necessary to produce and cultivate seedlings in a planned manner. However, conventional methods of germinating *T*. *vernicifluum* seeds generally result in a very low germination rate [6], and this is a major bottleneck in seedling production.

Dry seeds start germinating after absorbing water. Water absorption induces physiological and morphogenic alterations by causing large-scale changes at the transcriptomic, proteomic, and metabolomic levels [7–14]. Notably, the enhancement of gibberellic acid (GA) biosynthesis plays an important role in seed germination [15, 16]. GA is a phytohormone that promotes germination by activating embryo growth, cell wall loosening, and energy production [17–21]. However, the functioning of GA antagonizes abscisic acid (ABA; another phytohormone) signals, and deeply dormant seeds strongly suppress germination by accumulating high levels of ABA [17–21]. The seeds of several plant species remain dormant for a certain period until the ambient environment—such as temperature and light conditions—favors seedling growth. Overwintering seeds are usually deeply dormant; however, after experiencing prolonged cold in winter, the seeds acquire the ability to germinate through a process called vernalization. Vernalization has been studied in-depth in model plants (such as *Arabidopsis thaliana*), and studies have shown that cold treatment after water absorption promotes multiple germination-associated processes, including increased and decreased levels of endogenous GA and ABA, respectively [17–21]. Therefore, changes in GA and ABA levels and the sensitivities of seeds to these phytohormone levels are considered to be part of the central regulatory mechanisms underlying the maintenance and release of seed dormancy.

Seed dormancy is thought to be controlled not only by physiological but also by physical mechanisms [18]. Physical dormancy is mainly caused by the significant rigidity and water-impermeable nature of the endocarp. *Toxicodendron vernicifluum* belongs to the family Anacardiaceae, which also includes the poison oak (*T*. *diversilobum*), poison sumac (*T*. *vernix*), poison ivy (*T*. *radicans*), *Rhus aromatica*, *R*. *glabra*, *R*. *trilobata*, *R*. *virens*, and *R*. *typhina* [22, 23]. The seeds of most of these species are covered with a water-impermeable endocarp, have a very low germination rate, and remain in a physically enforced dormancy [24–26]. However, previous studies have shown that partial destruction of the pericarp with sulfuric acid enables water absorption, thereby significantly promoting seed germination [24–26]. Accordingly, *T*. *vernicifluum* seeds are treated with sulfuric acid or wood ash before sowing to promote germination, and these treatments have been incorporated into conventional seedling management [27]. However, the germination rate of *T*. *vernicifluum* seeds remains very low, often being below 10% [6]. Since *T*. *vernicifluum* seeds are formed in the fall and germinate in the spring, vernalization may affect their germination. Recent study has shown that cold treatment for a period of time improves seed germination of *T*. *vernicifluum* [28]. However, the physiological dormancy properties of *T*. *vernicifluum* seeds are unclear, as treatment of GA_3_, which is commonly used as a germination promoter, did not appear to be effective in breaking seed dormancy [29].

In this study, we investigated the seed germination characteristics of *T*. *vernicifluum*. Treatment with GA and cold temperatures for a specific duration effectively promoted the germination of sulfuric acid-treated seeds. Seed viability analysis revealed that only approximately 23.4% of seeds prepared by traditional methods had embryos capable of germination. Our findings suggest that the low germination rate of *T*. *vernicifluum* seeds is due to the deep dormancy of seeds (brought about by physical and physiological mechanisms) and the low viability of embryos. Based on our results, we propose the addition of two more steps to the traditional seedling treatment method—seed selection based on specific gravity (SG) and inactivation of seed dormancy—to improve seedling production.

## Materials and methods

### Seed collection

Approximately 70% of the urushi in Japan is produced in Iwate Prefecture [5], and Joboji Town in Ninohe City is one of the leading producers of urushi. Therefore, we investigated the germination characteristics of *T*. *vernicifluum* seeds collected in Joboji-machi. The seeds were collected in the “forest of cultural treasure” managed by Ninohe City (40°11′21.206′′S, 141°7′17.875′′E) during November in the years 2018–2020. The seeds were dried in a cool and dark place for more than 2 months. After threshing, the seeds were stored at 4°C until further analysis.

### Preparation of seeds for the germination assay

**Fig 1:**
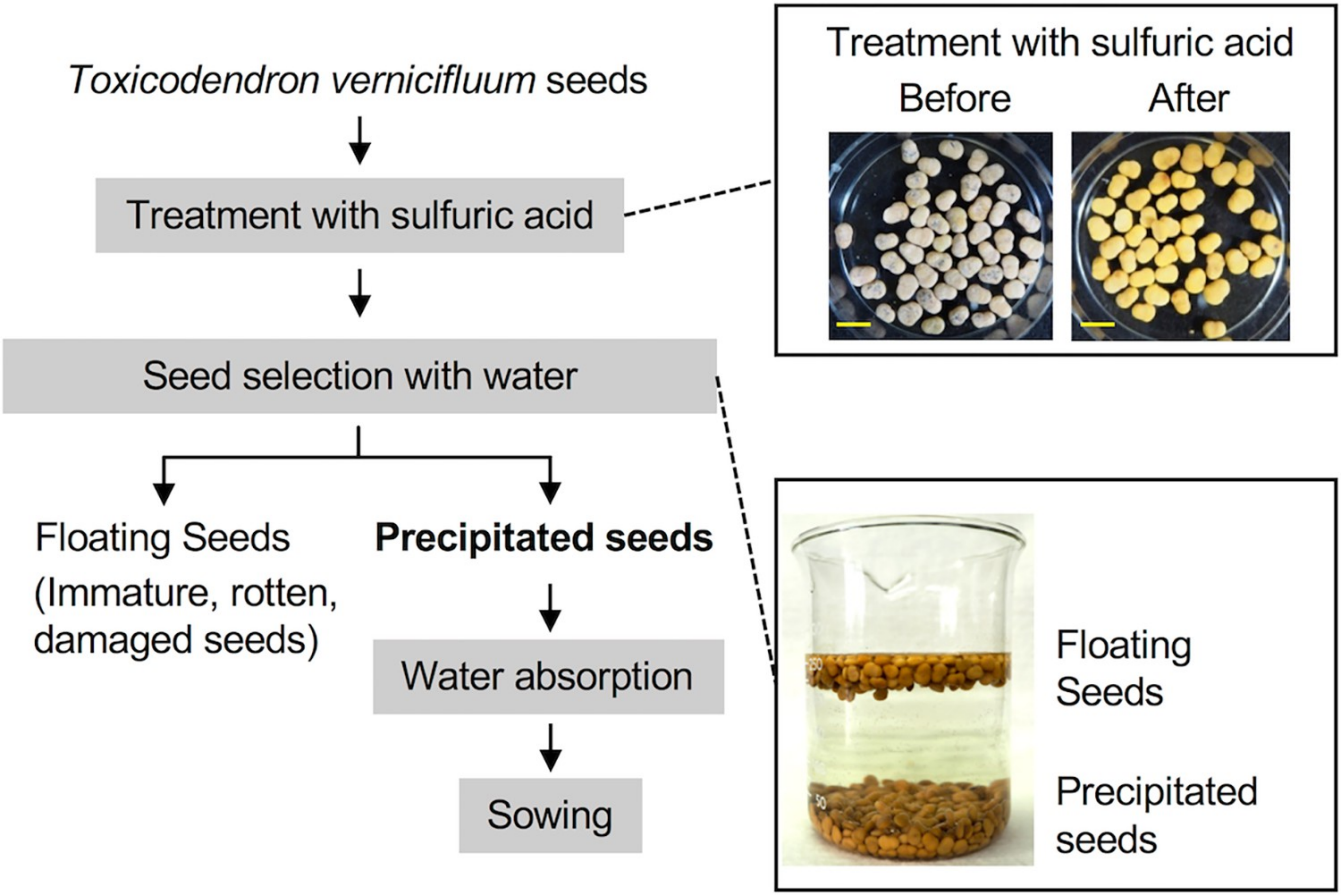
Schematic of seed preparation by conventional methods. Before sowing, the seeds are pretreated with sulfuric acid and then selected based on the differences in specific gravity. Photographs of the preparation process are shown. Scale bar = 1 cm.

Seeds were prepared according to the conventional method [27] (Fig 1). To increase the water permeability of the pericarp, 100 g of mature desiccated seeds (approximately 1000 seeds) were incubated with 200 mL sulfuric acid for 30 min with gentle stirring every 10 min. After the seeds were washed thoroughly with water, they were incubated in 1000 mL of water for 5 min. Following the conventional method, most of the seeds in the floating fraction were considered incapable of germinating, and only seeds in the precipitated fraction were used for subsequent processing. The seeds that precipitated were collected and soaked in sterile water for up to 7 days at room temperature (about 23 degrees). The seeds were examined to ensure that the endocarp had become translucent after absorbing water and then used for the germination assay.

### Germination assay

A sterilized filter paper was placed in a 90 mm Petri dish and moistened with 4 mL of water containing 0.2% Plant Preservative Mixture™ (Nakarai Tesque, Inc.). Approximately 50 seeds were placed on the filter paper, and the plates were covered and sealed to prevent drying. The plates were placed in a plant incubator and incubated under continuous light at 20°C for 60 days. For cold pretreatments, the sowed seeds were pre-incubated on moistened filter paper at 4°C in the dark for 0–4 weeks before being incubated under continuous light at 20°C for 60 days. For GA treatments, 100 mM stock solutions of GA_3_ and GA_4_ (Wako Pure Chemical Industries, Ltd.) were prepared in dimethyl sulfoxide (DMSO). Each stock solution was added to 4 mL of water containing 0.2% Plant Preservative Mixture™ to a final concentration of 150 μM GA_3_, GA_4_ or 0.15% DMSO (Mock). Approximately 50 seeds were placed on a filter paper moistened with GA solution and incubated for 60 days. Seed germination rate (as determined by radicle protrusion) was measured 60 days after the start of incubation for all treatment groups. At least 50 seeds were tested in each independent experiment, and data were obtained from at least three biological replicates (*n* = 3).

### Seed selection based on seed SG

Following the aforementioned sulfuric acid treatment, the seeds were placed in 1000 mL of water to separate the floating and precipitated seeds. The floating seeds corresponded to an SG of < 1.00. The precipitated seeds were sequentially added to sodium chloride solutions of 1.07, 1.15, and 1.19 SG, and the seeds that floated in each solution were separated. Finally, the seeds were classified into five groups with different SGs: SG < 1.00, 1 ≤ SG < 1.07, 1.07 ≤ SG < 1.15, 1.15 ≤ SG < 1.19, and SG ≥ 1.19. All seeds were thoroughly rinsed with water and used in the subsequent experiments.

### Seed viability test

Seed viability was examined using 2,3,5-triphenyl tetrazolium chloride (TTC) staining of embryos [30]. After the aforementioned sulfuric acid treatment, seeds were soaked in sterile water for up to 7 days. Next, the seeds that had absorbed water were cut through the center with a scalpel, and the embryos were collected. Approximately 50 embryos were submerged in 0.2% (w/v) TTC (Sigma-Aldrich) solution and incubated at 30°C for 24 h in the dark. The stained embryos were imaged after incubation, and the viable embryos were counted. Data were obtained from three biological replicates (*n* = 3).

### Statistical analysis

The quantitative values presented in the figures were obtained using GraphPad Prism 8 and Microsoft Excel using basic inbuilt statistical functions. Datasets were analyzed using one-way analysis of variance (ANOVA) with Tukey’s multiple comparisons test, the two-tailed unpaired *t*-test, or the Kruskal–Wallis test with post-hoc Dunn’s multiple comparisons test. The results of statistical significance tests are included in the legend of each figure, and *n* represents the number of independent experiments performed for each test. Pearson’s correlation coefficient was calculated using Microsoft Excel. The minimal dataset of this study has been uploaded as S1 Data.

### Ethics statement

The study area belongs to the Iwate Prefectural Forest Technology Center. Permits for *T*. *vernicifluum* seed collection on the field were obtained (verbal approval) from the Iwate Prefectural Forest Technology Center. Seed collection was accompanied by staff belong to the Iwate Prefectural Forest Technology Center. This study did not require ethical permission as no endangered or protected species, animals, or destructive sampling were involved.

## Results

### Inactivating seed dormancy in *T*. *vernicifluum* with GA_4_ and cold temperature treatment

**Fig 2:**
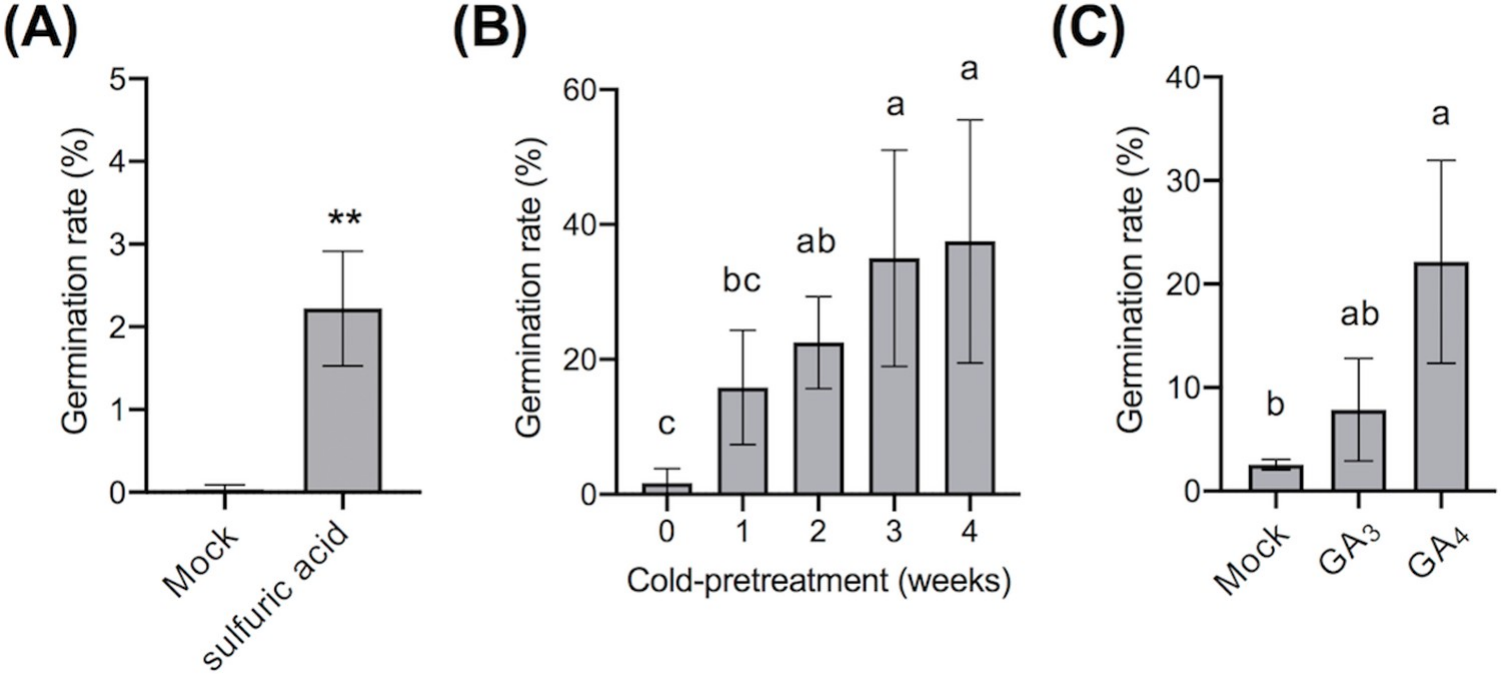
Effect of gibberellic acid and cold pretreatment on the germination of *Toxicodendron vernicifluum* seeds. **(A)** Germination-promoting effect of sulfuric acid on *T*. *vernicifluum* seeds. The germination rate represents the average of three independent experiments (*n* = 3). The control was treatment with water (Mock). **(B)** Cold pretreatment promotes germination in *T*. *vernicifluum* seeds. Before incubation at 20°C, the seeds were exposed to cold temperature (4°C) for 0–4 weeks. Cold-treated seeds were incubated at 20°C, and germinated seeds were counted 60 days after the start of incubation. The germination rate represents the average of nine independent experiments (*n* = 9). **(C)** Germination-promoting effects of GA_3_ and GA_4_. The control was treatment with 0.15% DMSO (Mock). The germination rate represents the average of three independent experiments (*n* = 3). Significant differences (**P < 0.01) were determined by with an unpaired *t*-test with Welch’s correction (A). Datasets were analyzed using the Kruskal–Wallis test with post-hoc Dunn’s multiple comparisons test (B) or one-way ANOVA with Tukey’s multiple comparisons test (C) with a significance level of 5% (*P* < 0.05).

We first examined the promoting effect of sulfuric acid on *T*. *vernicifluum* seed germination. Precipitated seeds were selected based on their SG in water, treated with sulfuric acid, and soaked in water for 7 days to allow water absorption. Following this, the seeds were placed on sterile damp filter paper and incubated. Seeds that were not treated with sulfuric acid did not germinate (Mock in Fig 2A); however, germination was observed in a low proportion (2.6 ± 0.5%) of seeds that were treated with sulfuric acid (Fig 2A). Next, we exposed the selected seeds to low temperatures (4°C) for 1–4 weeks and measured their germination rate. Cold pretreatment for at least 2 weeks significantly promoted seed germination (21.1 ± 6.3%) compared to control (as 0 week) conditions (1.3 ± 1.7%), and cold pretreatment for more than 3 weeks was the most effective in this regard (35.5 ± 16.0%) (Fig 2B).

Next, we tested whether treatment with GAs promotes the germination of *T*. *vernicifluum* seeds. GAs comprise a group of over 100 structurally related compounds containing an ent-gibberellane skeleton. However, only some GAs have biological activities; these include 13-hydoxy GAs (such as GA_1_ and GA_3_) and non-13-hydoxy GAs (such as GA_4_ and GA_7_) [31]. In agriculture, GA_3_ is widely used to promote seed germination, stem elongation, flowering, fruit maturation, and the production of seedless fruits. However, the effects of GA treatment often depend on the plant species and GA isoform [32, 33]. In this study, we tested the effects of both GA_3_ and GA_4_ on *T*. *vernicifluum* seed germination. GA_3_ treatment promoted seed germination (7.8 ± 5.0%), but the germination rate was not significantly different from that in the Mock treatment group (2.7 ± 1.1%). In contrast, GA_4_ treatment significantly promoted the germination of *T*. *vernicifluum* seeds (22.2 ± 9.8%), resulting in a germination rate that was approximately 10 times higher than that of the Mock treatment (Fig 2A). GA_4_ is often not commercially available for pesticides, and cold treatment is more practical for seedling management. Therefore, we applied cold pretreatment to the seeds in subsequent germination tests.

### Effects of SG on seed germination in *T*. *vernicifluum*

Compared to control conditions, cold pretreatment and the application of GA_4_ promoted the germination of *T*. *vernicifluum* seeds (Fig 2). However, the improved germination rate was only 20–35%, and > 50% of the treated seeds failed to germinate. These results suggested that either seed dormancy was not completely inactivated by these treatments, or that only a small number of seeds were capable of germination. Several crop species exhibit a close relationship between seed maturity and SG [34]. Therefore, we estimated the germination rate of seeds based on their SG in various sodium chloride solutions. Because seed quality varies from year to year, we also conducted a 3-year follow-up survey to confirm the reproducibility of the data.

**Fig 3:**
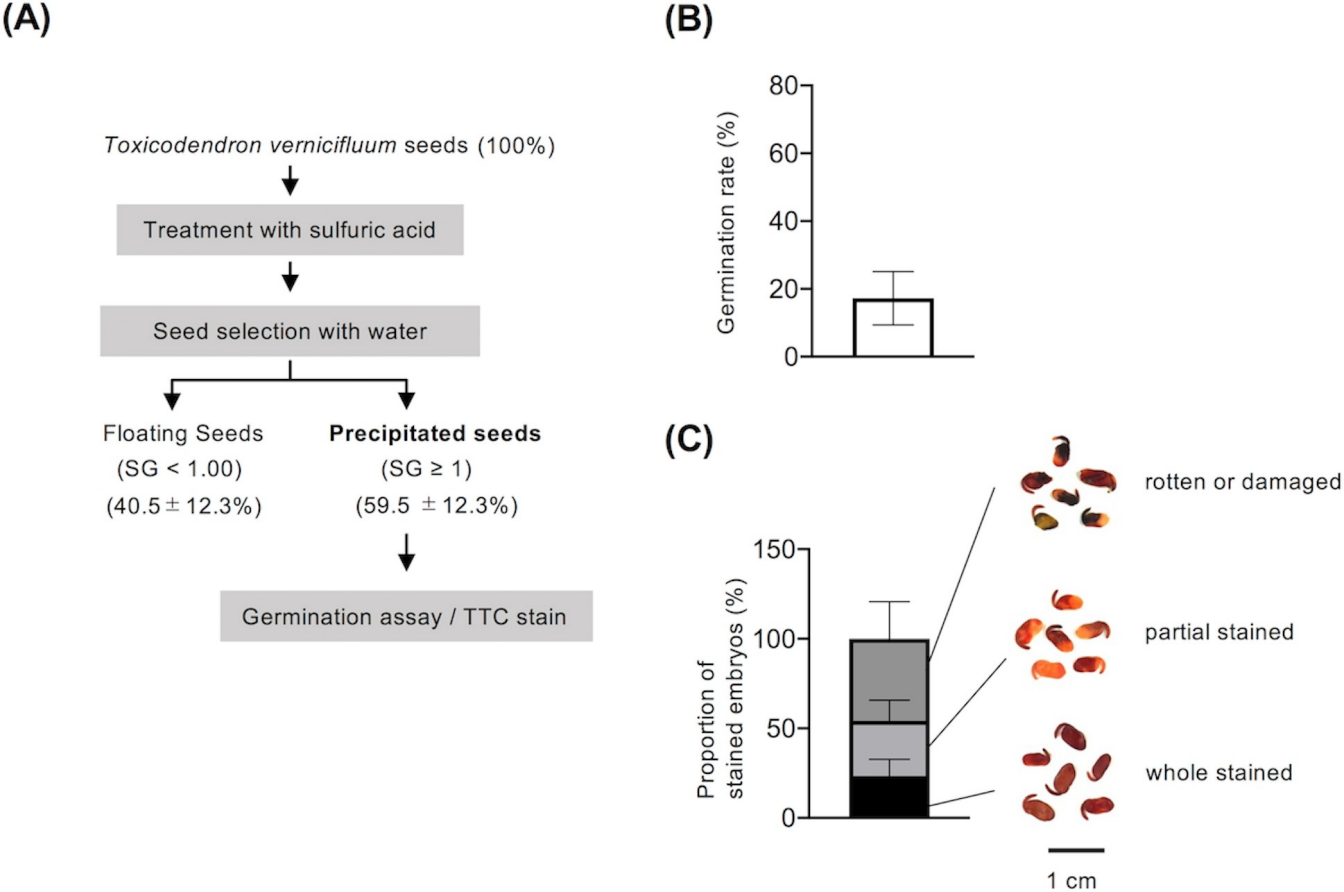
Germination characteristics and viability of seeds selected by conventional methods. **(A)** Schematic of seed selection by conventional methods. Seeds were separated using sodium chloride solutions of different specific gravities (SGs), and the proportions (%) were calculated based on the initial number of seeds. **(B)** Germination rate. After cold pretreatment for 3 weeks, seeds were incubated at 20°C, and germinated seeds were counted 60 days after the start of incubation. **(C)** Seed viability test. Seeds were cut with a sharp scalpel to collect embryos. The embryos were incubated overnight with TTC solution and photographed (right). The stained embryos are classified into three types: whole-stained embryos, partially stained embryos, and rotten or damaged embryos (right). The total number of embryos investigated was normalized to 100, and the proportion of embryos of each type was calculated (left). Averages and standard deviations are shown for seeds collected in 2018, 2019, and 2020 (*n* = 3) (B, C). Scale bar = 1 cm.

Following the conventional method, we first added sulfuric acid-treated seeds to water and separated them into floating and precipitated fractions. The seeds in the floating fraction were classified into grades based on their SG. Those with an SG of < 1 accounted for approximately 40% of the total seeds (Fig 3A). These low-SG seeds do not germinate, and are not used for conventional sapling production [27]. In the germination test with cold pretreatment, seeds with an SG ≥ 1 had a germination rate of 17.3% ± 7.9% (Fig 3B). The seed viability test with TTC staining showed that whole-stained embryos accounted for only 23.4% ± 9.2% of all tested seeds with SG ≥ 1 (Fig 3C).

**Fig 4:**
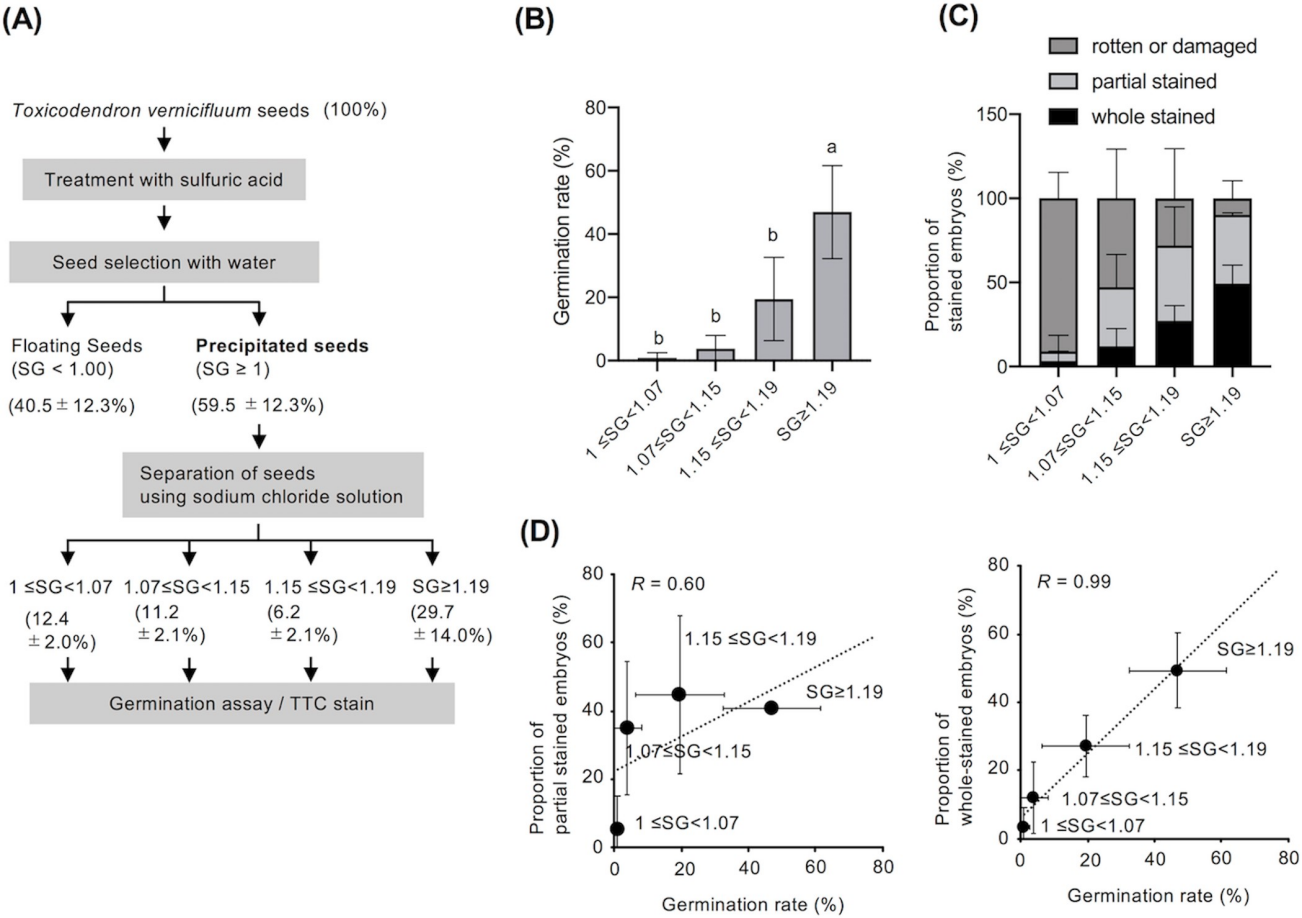
Relationship between seed specific gravity (SG) and germination ability. (A) Proportion of seeds with different SGs. Seeds were separated using sodium chloride solutions of different SGs, and the proportions (%) were calculated based on the initial number of seeds. (B) Effect of seed SG on germination. After cold pretreatment for 3 weeks, seeds were incubated at 20°C, and germinated seeds were counted 60 days after the start of incubation. Averages and standard deviations are shown for seeds collected in 2018, 2019, and 2020 (*n* = 3). Datasets were analyzed using one-way ANOVA with Tukey’s multiple comparisons test with a significance level of 5% (*P* < 0.05). (C) Relationship between the SG of seeds and the proportion of seeds with mature embryos. TTC-stained embryos are classified into three types: whole-stained embryos, partially stained embryos, and rotten or damaged embryos. The total number of embryos investigated was normalized to 100, and the proportion of embryos of each type was calculated. (D) Correlation between the percentage of partially stained or whole-stained embryos in seeds and the germination rate. *R* indicates the calculated Pearson’s correlation coefficient. Averages and standard deviations are shown for seeds collected in 2018, 2019, and 2020 (*n* = 3) (C, D).

Next, we used sodium chloride solutions of different SGs to obtain four grades of seeds with different SGs (Fig 4A). Seeds with SG > 1 were subjected to cold pretreatment (4°C) for 3 weeks before incubation. The results of this test showed that the germination rate increased with increasing SG of the seeds, and seeds with SG ≥ 1.19 showed the highest germination rate (46.9% ± 14.7%) (Fig 4B). In addition, the proportion of whole-stained embryos increased with increasing seed SG (Fig 4C). In contrast, the proportion of rotten or damaged seeds showed an inversely proportional relationship with seed SG (Fig 4C). Correlation analysis revealed a high correlation between the proportion of whole-stained embryos and the germination rate (*R* = 0.99) (Fig 4D), suggesting that embryos that were entirely stained by TTC had higher germination potential than partially stained or unstained embryos.

## Discussion

Urushi has long been used in traditional handicrafts, and is highly valued for its cultural and artistic value [1]. To protect traditional urushi culture and ensure its inheritance by future generations, it is essential to grow the trees that produce urushi. However, *T*. *vernicifluum* seeds rarely germinate under normal conditions, which hinders planned seedling production [6]. This study demonstrates that pretreatment with GA_4_ rather than GA_3_ significantly promotes the germination of *T*. *vernicifluum* seeds (Fig 2C), suggesting that seeds have different susceptibility to GA isoforms. Furthermore, cold pretreatment was particularly effective in improving seed germination rates (Fig 2B). Taken together, these results suggest that *T*. *vernicifluum* seeds are maintained in a deeply dormant state by physiological mechanisms, and that seed germination is controlled by vernalization involving GA signals. In addition, the hard and water-impermeable endocarps of *T*. *vernicifluum* seeds had to be decomposed with sulfuric acid to ensure efficient germination. A morphological study of *R*. *aromatica* seeds revealed that sulfuric acid erodes the brachysclereids and osteosclereids in the carpellary micropyle region, thus inducing water absorption [24]. Sulfuric acid treatment likely has a similar effect on *T*. *vernicifluum* seeds. Our results suggest that both physiological and physical mechanisms are involved in the dormancy of *T*. *vernicifluum* seeds, which is consistent with findings in other plants in the Anacardiaceae family [24, 25]. The seeds of several species with physical dormancy are known to exhibit extremely high cold tolerance. In addition, the water-impermeable endocarps of dried seeds protect embryos from freezing by blocking the ingress of water from the outside environment [35]. Iwate Prefecture—the main producer of urushi in Japan—experiences a lot of snowfall, and temperatures often fall below minus 10 degrees. Therefore, it is likely that both physiological and physical mechanisms play a role in increasing seed viability and preventing germination in unsuitable environments.

The analysis of seed viability and germination ability revealed that more than half of the seeds produced by *T*. *vernicifluum* were empty or had embryos that were incapable of germination (Fig 3). A 3-year follow-up study revealed a high correlation between the proportion of viable embryos and germination rate (Fig 3). Since *T*. *vernicifluum* seeds contain very little endosperm, the primary nutrients needed for germination and seedling growth are probably stored in the cotyledons or hypocotyls [23]. Therefore, the physiological state of the embryo can have a significant effect on germination efficiency. Taken together, these results suggest that in addition to seed dormancy, the low proportion of intact mature seeds is also responsible for the low germination rate of *T*. *vernicifluum* seeds.

In conclusion, we demonstrate that selecting seeds based on their SG is effective for harvesting seeds with a higher germination ability (Fig 4). This approach may help stabilize the production efficiency of *T*. *vernicifluum* seedlings, reduce labor costs, and help eliminate differences in seed quality between years. To improve the low germination rate of *T*. *vernicifluum* seeds, we propose adding two more steps to the conventional method of sulfuric acid treatment: (1) selecting seeds with an SG greater than 1.15 or 1.19; and (2) inactivating physiological dormancy through cold pretreatment for approximately 3 weeks. Sulfuric acid is effective for the partial decomposition of the endocarp, but may have adverse effects on the human body and the environment. Therefore, future studies should aim at developing new endocarp treatment methods that can alleviate the problems with sulfuric acid treatment.

## Supporting information

S1 Data Minimal dataset used in the study. (XLSX)
